# Chemical and microbial quality of bottled drinking water in Gonabad city, Iran: Effect of time and storage conditions on microbial quality of bottled waters

**DOI:** 10.1016/j.mex.2019.02.001

**Published:** 2019-02-05

**Authors:** Mahmoud Shams, Mehdi Qasemi, Mojtaba Afsharnia, Alireza Mohammadzadeh, Ahmad Zarei

**Affiliations:** a Department of Environmental Health Engineering, Faculty of Health, Mashhad University of Medical Sciences, Mashhad, Iran; b Social Determinants Health Research Center, Mashhad University of Medical Sciences, Mashhad, Iran; c Department of Environmental Health Engineering, Faculty of Health, Social Development and Health Promotion Research Center, Gonabad University of Medical Sciences, Gonabad, Iran; d Department of Microbiology, Faculty of Medicine, Gonabad University of Medical Sciences, Gonabad, Iran

**Keywords:** Microbial quality, Chemical quality, Bottled water

## Abstract

Reports from bottled water (BW) industries show a rapidly increasing rate of global bottled water consumption. The present paper contains data on chemical and microbial quality of bottled waters marketed in Gonabad city, Iran. The data on the effect of time and storing conditions on microbial quality of bottled water also is reported. The physical and chemical parameters of all 9 studied BW brands meet well with those mentioned on the labels. All BW sampled also were free of pathogenic indicators (total coliforms, fecal coliforms, fecal streptococci and clostridium perfringens). BWs kept in refrigerator have minimal heterotrophic and pathogenic bacterial count. The highest bacterial count was observed when BWs were exposed to indirect sunlight at room temperature. Presence of heterotrophic and especially pathogenic bacteria reduced significantly when the samples were placed to direct sunlight. In all samples, apart from where they were kept, the heterotrophic and pathogenic bacterial counts showed an increasing trend after bottling.

## Graphical abstract

**Figure:**
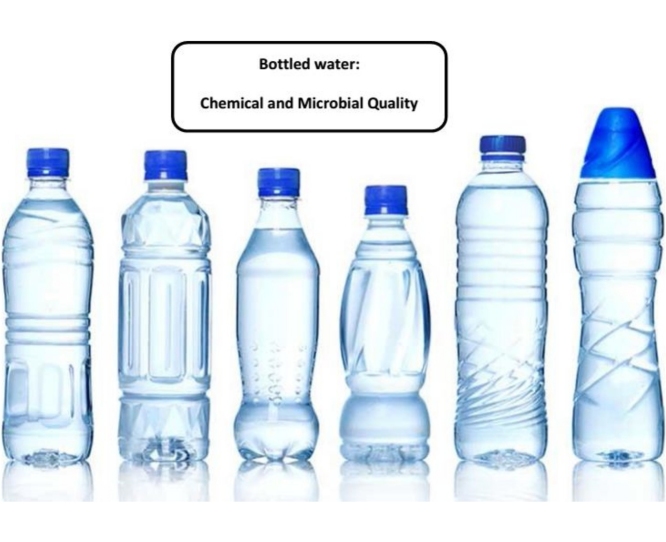

**Specifications Table**

**Table:** 

**Subject area**	Chemistry, Biology
**More specific subject area**	Drinking water, bottled water
**Type of data**	Table, graph
**How data was acquired**	The chemical parameters were determined using a Spectrophotometer (DR-5000 Spectrophotometer, USA) Microbial parameters of bottled water were determined by heterotrophic plate count (milk agar, blood agar), P/A, MPN
**Data format**	Raw, analyzed
**Experimental factors**	Bottled waters kept in different conditions (refrigerator, direct/indirect exposure to sunlight as well as darkness) were used to collect data on the effect of time and storage condition on heterotrophic and pathogenic bacterial count.
**Experimental features**	Very brief experimental description
**Data source location**	Gonabad, Razavi Khorasan province, Iran.
**Data accessibility**	All data included in the article.

**Protocol data**	
• While all countries are experiencing growing trend of bottled water consumption, it is necessary to know the effect of storing condition on its microbial quality.	
• The heterotrophic and pathogenic bacterial counts increased since bottling of bottled water.	
• While keeping bottled water in refrigerator (3-5 °C) could minimize the bacterial count, the highest bacterial growth occurred when they expose to indirect sunlight at room temperature.	
• Heterotrophic and especially pathogenic bacteria reduced significantly in the presence of direct sunlight.	
• The physical and chemical parameters of BWs marketed in Gonabad meet well with those mentioned on the labels and also were free of pathogenic indicators (total coliforms, fecal coliforms, fecal streptococci and clostridium perfringens).	

## Description of protocol

Rapid population growth, urbanization and industrialization have led to the pollution of many water resources worldwide [[1], [2], [3], [4]]. Bottled water is widely accepted as a reliable healthy drinking water in many parts of the world including Iran [[5], [6], [7]]. Therefore, investigation of bottled water quality is of prime importance. This article presents data collected from bottled waters with the aim of evaluating the chemical and microbial quality of these waters. The chemical composition of the samples was summarized in Table 1. Blood agar was applied as a medium for enumeration of pathogenic bacteria, heterotrophic bacteria counts was done in a R2A agar medium. A colony counter was utilized for bacterial enumeration and the number of colonies was reported as cfu/mL.

**Table 1:** Characteristics of nine brands of bottled water (information on label of bottle).

Brand	pH	Percent difference	F	Percent difference	NO_3_	Percent difference	Ca	Percent difference	Mg	Percent difference	SO_4_	Percent difference	Cl	Percent difference	K	Percent difference	Na	Percent difference	TH	Percent difference
**A**	7.2	4.1	0.2	5	1.8	5.7	78.5	7.9	18.7	−5.9	20	5.1	N.A	N.A	0.8	18.1	6.2	1.8	N.A	N.A
**B**	7.5	3.2	0.23	−9.5	4.04	−2.5	62.7	7.2	20.3	10.2	20.9	−21.1	16.4	32.4	1.37	30.6	10.8	13.7	N.A	N.A
**C**	7.8	0.2	0.15	0.86	N.A	N.A	56	15.5	13.15	−5.3	18	16.2	25	12.5	N.A	N.A	0.65	22.1	180	13.8
**D**	7.8	−1.2	0.1	−2.5	6.4	−8.1	36	−5.5	16.8	6.3	19.2	6.7	7.1	62.2	N.A	N.A	11.5	−12.3	N.A	N.A
**E**	7.3	3.1	<0.2	0.4	<7	N.A	28	−7.5	22	−7.1	10	1.9	6.9	54.3	1	35.2	6	−4.9	90	−2.6
**F**	7.5	5.3	N.A	N.A	N.A	N.A	41.6	1.8	42.7	7.2	30	−7.3	74.4	7.1	N.A	N.A	78	−17.7	145	−19.4
**G**	7.6	2.6	0.1	−2.8	5.5	9.4	60	0.9	12	N.A	34	9.1	12.2	−11.1	1.2	18.4	21	−6.8	N.A	N.A
**H**	7	−3.9	0.36	6−	3.7	−4.8	32.3	−6.4	10.8	N.A	30	−4.6	18.5	−5.6	N.A	N.A	N.A	N.A	N.A	N.A
**I**	N.A	N.A	0.2	0.5	N.A	N.A	N.A	N.A	N.A	N.A	N.A	N.A	N.A	N.A	N.A	N.A	N.A	N.A	N.A	N.A
**Ave**	7.46	3.8	0.19	1.69	4.28	7.46	49.4	6.7	19.5	7.9	22.7	5.7	22.9	33.7	1.09	25.5	19	12.5	138.3	13.8
**Min**	7	0.2	0.1	0.4	1.8	7	28	0.9	10.8	6.3	10	−4.6	6.9	7.1	0.8	18.1	0.6	1.8	90	26−
**Max**	7.8	5.3	0.36	5	6.4	7.8	78.5	15.5	42.7	10.2	34	16.2	74.4	62.2	1.37	35.2	78	22.1	180	13.8

N.A: Not Available, TH: Total hardness, Ave: Average, Min: Minimum, Max: Maximum.

**Fig. 1:**
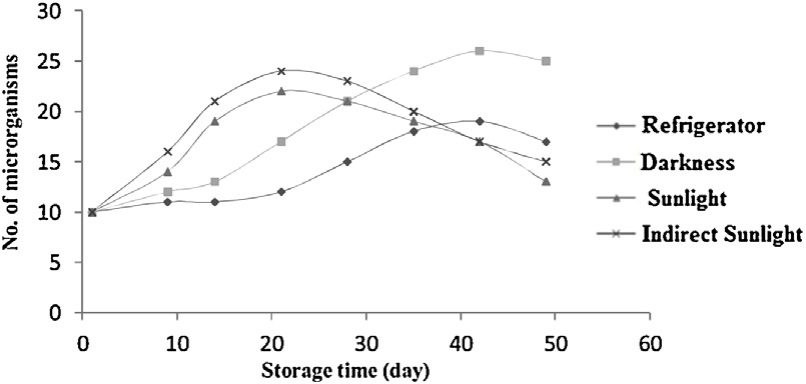
Changes in the number of heterotrophic bacteria in bottles during time based on storage conditions.

**Fig. 2:**
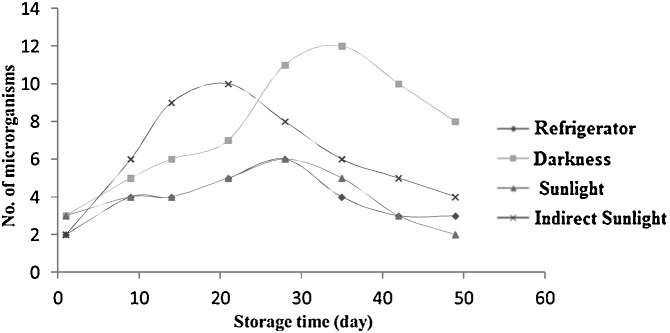
Changes in the number of pathogenic bacteria in bottles during time based on storage conditions.

Fig. 1 illustrates the change in heterotrophic bacterial population at R2A agar medium during storage. Fig. 2 depicts the change in pathogenic bacterial population at blood agar medium. The results of presence absence analysis (P/A) for coliforms, fecal streptococcus, and most probable number (MPN) for fecal coliforms are given in Table 2.

**Table 2:** The data of presence absence analysis (P/A) for coliforms, fecal streptococcus, and most probable number (MPN) for fecal coliforms.

MPN	Fecal streptococcus	P/A	Brand
0	<2	25%	A
0	<2	33.3%	B
0	<2	0	C
0	<2	25%	D
0	<2	0	E
0	<2	0	F
0	<2	50%	G
0	<2	0	H
0	<2	29%	I

Fecal streptococcus analysis was conducted based on method 9230 of standard methods for the examination of water and wastewater [8]. Formation of dark colonies was the sign of the presence of fecal streptococcus. For clostridium perfringens, milk agar medium was used. For MPN, the samples were incubated at 35 °C for 3–5 days and the findings were reported as MPN/100 mL [9]. In his technique, McConkey's Lactose Bile Salt Broth with Bromocresol purple as an indicator and Brilliant Green Lactose Bile Broth (BGLB) were used. For estimation the presence or absence of coliforms, the tubes were incubated at 35 °C for 48 h [10]. A pH meter model (WTW 340i) was used for the measurement of pH of samples.

For hardness measurement, titration method using EDTA was utilized. Chloride was determined using argentometric titration. Sodium (Na) and potassium (K) was determined using a flame photometer (Jenway Co.). Nitrate was measured using ultraviolet spectrophotometric screening procedure [11]. Sulfate was measured using Turbidimeter (UNICO), and fluoride was measured SPADNS spectrophotometric method [12].

### Sampling and analysis

This cross-sectional study was conducted for 9 mostly used brands of bottled waters (BWs) sold in Gonabad markets in Iran. These brands were selected randomly from 15 brands in Gonabad’s markets. Both chemical and microbial qualities of bottled waters were examined. Moreover, the effects of time, storage conditions as well as temperature were on the microbial quality were studied. All the studied brands were non-carbonated water. The volume of water bottles used in the tests were 500 cc. Microbial examinations included enumeration of HPC and coliform bacteria as well as testing for presence or absence of coliforms during two months of storage.

All the measurements were conducted according to Standard Methods for the Examination of Water and Wastewater. For the microbial study, four different storage conditions including storage in refrigeration (4 °C), in presence of sunlight (25 °C), in indirect sunlight (20 °C) and darkness (20 °C) were examined. The microbial samplings were conducted once a week during two months storage.
